# Polyomavirus Drives Cerebellar Lesions in AIDS Monkeys With Ataxia

**DOI:** 10.1002/mco2.71049

**Published:** 2026-10-19

**Authors:** Tian‐Zhang Song, Li‐Rong Zhang, Yun‐Mei Wang, Yan Hu, Qing Li, Ren‐Rong Tian, Liu‐Meng Yang, Si‐Dong Xiong, Dong‐Dong Wu, Yong‐Tang Zheng

**Affiliations:** ^1^ State Key Laboratory of Genetic Evolution & Animal Models National Resource Center For Non‐Human Primates Kunming Institute of Zoology Chinese Academy of Sciences Kunming China; ^2^ KIZ‐SU Joint Laboratory of Animal Model and Drug Development College of Pharmaceutical Sciences Soochow University Suzhou China; ^3^ University of Chinese Academy of Sciences Beijing China; ^4^ Yunnan Provincial Key Laboratory of Entomological Biopharmaceutical R&D College of Pharmacy Dali University Dali China

**Keywords:** AIDS, ataxia, nonhuman primate model, polyomavirus, SV40

## Abstract

Cognitive and motor dysfunction occurs in 30%–50% of people with HIV/AIDS. Within this clinical spectrum, HIV‐associated ataxia is an uncommon but debilitating disorder with poorly defined neuropathological mechanisms. This study investigated two rhesus macaques with chronic simian immunodeficiency virus (SIV) infection that developed progressive ataxia, characterized by postural imbalance and nystagmus. Comparative neuropathological profiling of cerebellar tissue from ataxic SIV‐infected macaques, non‐ataxic SIV‐infected macaques, and uninfected controls revealed that ataxia was associated with diffuse cerebellar atrophy, disrupted tissue organization, Purkinje cell degeneration, demyelination, glial nodule formation, and prominent microglial neuroinflammation. Cerebellar infection with simian polyomavirus 40 (SV40), a member of the *Polyomaviridae* family, was identified as the cause of the lesions, with viral tropism primarily involving cerebellar granule neurons. Mechanistically, SV40 infection induced neuronal pyroptosis and inflammatory cytokine release, promoting microglial recruitment and activation, amplifying local neuroinflammation, and culminating in nodular aggregate formation. Collectively, these findings establish SV40 infection as a direct driver of cerebellar pathology in SIV‐associated ataxia and define a pathogenic sequence linking granule neuron pyroptosis, microglial activation, and progressive cerebellar injury—a cascade that may parallel polyomavirus‐induced ataxia in people living with HIV‐1.

## Graphical abstract

Chronic SIV‐infected macaques with ataxia develop severe cerebellar lesions driven by simian polyomavirus 40 (SV40) infection. SV40 preferentially infects cerebellar granule cells, inducing pyroptosis and an aberrant proliferative state. Pyroptotic neuronal injury promotes microglial recruitment and activation, amplifying local neuroinflammation and culminating in microglial nodule formation (created in BioRender. S, T. (2026); https://BioRender.com/g8qaugd).

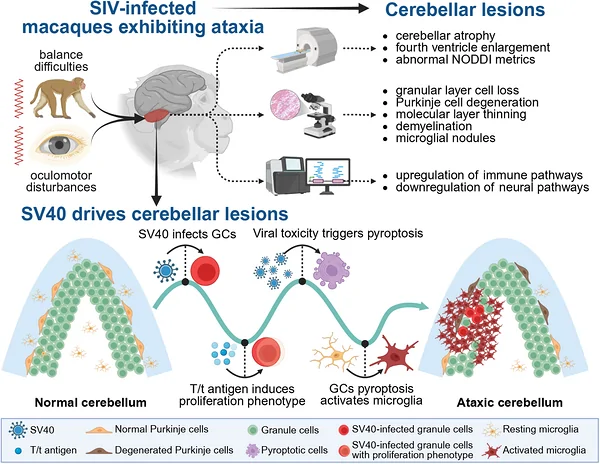

## 1 Introduction

Neurological disease in people living with HIV (PLWH) is clinically heterogeneous, but many patients develop overlapping cognitive and motor abnormalities, including deficits in attention, memory, executive function, and information processing, as well as gait disturbance, limb weakness, tremor, and ataxia [1, 2]. Cognitive impairment, reported in approximately 39.6% of PLWH, has been studied extensively under the framework of HIV‐associated neurocognitive disorder (HAND), with emphasis on the prefrontal cortex, hippocampus, striatum, and related cognitive circuits [3, 4]. In contrast, the neurobiological basis of motor dysfunction remains less clearly defined, although motor symptoms frequently coexist with cognitive impairment and may arise from related neural injury. This gap is particularly relevant to the cerebellum, a structure required for motor coordination that also contributes to cognitive processing [5, 6].

Ataxia is a cerebellar syndrome defined by impaired motor coordination and typically presents with gait instability, dysarthria, and oculomotor dysfunction [7]. Its association with HIV‐1 infection has been recognized since early case reports in 1998 [8], and a recent systematic review spanning 1987–2022 identified 148 published cases of ataxia in PLWH, 82.4% of which were associated with adverse clinical outcomes [2]. Cerebellar abnormalities were detected on magnetic resonance imaging (MRI) in all cases, and postmortem studies consistently showed granule cell loss, axonal swellings, and white matter degeneration, establishing cerebellar injury as the anatomical basis of HIV‐associated ataxia [2, 8]. However, the etiological landscape of HIV‐associated ataxia is multifactorial and differs by clinical context. In untreated HIV infection, progressive multifocal leukoencephalopathy (PML), a devastating demyelinating disorder of central nervous system (CNS) white matter caused by John Cunningham virus (JCV), remains the most frequently recognized specific etiology of HIV‐associated ataxia [2]. In the combination antiretroviral therapy (cART) era, treatment‐related neurotoxicity has emerged as an additional contributor to the burden of ataxia. Efavirenz, a non‐nucleoside reverse transcriptase inhibitor, has been implicated in delayed‐onset ataxia and encephalopathy that improve after drug withdrawal [9, 10]. Protease inhibitor‐associated ataxia has been reported primarily in the context of drug–drug interactions, and one reversible case has been attributed to raltegravir, an integrase strand transfer inhibitor (INSTI) [2, 11].

In PLWH not receiving cART, JCV can cause lytic infection of oligodendrocytes, producing the white matter demyelination characteristic of PML [12, 13]. However, JCV neuropathology is not restricted to white matter disease. The virus also exhibits distinct cerebellar tropism, directly infecting and destroying cerebellar granule cells (GCs) and producing a specific granule cell neuronopathy characterized by cerebellar atrophy in the absence of PML‐related white matter lesions [14, 15]. Critically, JCV‐associated GC injury has been observed in PLWH independently of a PML diagnosis, suggesting that its contribution to cerebellar pathology may be underrecognized [16]. Although JCV‐associated cerebellar degeneration is now recognized as an important substrate of HIV‐associated ataxia, the pathological sequence that links polyomavirus infection to cerebellar damage remains insufficiently defined. This uncertainty limits mechanistic interpretation and hinders the development of targeted therapeutic strategies.

Simian polyomavirus 40 (SV40), a small, non‐enveloped DNA virus of the *Polyomaviridae* family, provides a biologically relevant macaque counterpart for modeling polyomavirus‐associated neurological disease. JCV and SV40 are closely related polyomaviruses with parallel host‐restricted biology and overlapping neuropathogenic potential. Although JCV primarily infects humans and SV40 predominantly infects macaques, both viruses can persist latently in immunocompetent hosts and cause severe demyelination and other neuropathological lesions under immunosuppression [17]. A previous large‐scale serological survey of 1579 macaques across nine species revealed widespread SV40 seropositivity in *Macaca mulatta*, *Macaca fascicularis*, and *Macaca tonkeana* [18]. Seropositivity was highest overall in *M. mulatta* (74.7%) and was particularly common among animals imported from China, reaching 95% in *M. fascicularis* and 92.1% in *M. mulatta*. These findings indicate that SV40 infection is highly endemic in Chinese‐origin macaques. Notably, in simian immunodeficiency virus (SIV)‐infected macaques, SV40 recapitulates key features of JCV pathogenesis in PLWH, including fatal demyelination and spontaneous neuronal infection [19, 20]. These observations highlight the value of SIV‐infected nonhuman primate models for investigating how polyomavirus infection induces cerebellar lesions during AIDS‐associated ataxia. In the present study, two chronically SIV‐infected macaques not treated with cART developed unexpected progressive ataxia. Comparative neuropathological analysis identified cerebellar SV40 infection as a direct driver of the observed cerebellar lesions, with viral tropism primarily involving cerebellar granule neurons. These findings establish an etiological link between SV40 infection and SIV‐associated cerebellar ataxia and provide an experimental model for defining how polyomavirus infection triggers granule cell injury, microglial activation, and progressive cerebellar degeneration.

## 2 Results

### 2.1 Marked Cerebellar Lesions in SIV‐Infected Macaques With Ataxia

Seven adult Chinese rhesus macaques were assigned to three groups: uninfected controls (CTL; SIV^−^Ataxia^−^, *n* = 3; CTL1, CTL2, and CTL3), SIV‐infected macaques without ataxia (SIVn; SIV^+^Ataxia^−^, *n* = 2; SIV1 and SIV2), and SIV‐infected macaques with ataxia (SIVa; SIV^+^Ataxia^+^, *n* = 2; SIV3 and SIV4). Cohort information is summarized in Table S1. All SIV‐infected macaques belonged to the same cohort and shared the core features of chronic SIVmac239 infection, including 3.5 years of infection, persistent viremia, inverted CD4/CD8 ratios, and the absence of overt AIDS‐defining clinical symptoms such as cachexia or chronic diarrhea. The SIVa group was distinguished by sustained ataxia, manifested by persistent postural imbalance and nystagmus for at least 1 month (Videos S1 and S2).

Histological examination revealed a severity gradient in cerebellar pathology, with the most profound lesions observed in SIVa animals, milder abnormalities in SIVn animals, and minimal changes in CTL animals (Figure 1A). Ataxic macaques exhibited severe disruption of the granular layer and cerebellar white matter, accompanied by marked granule cell loss (27.4% lower than CTL and 15.0% lower than SIVn), increased Purkinje cell degeneration characterized by dendritic retraction and nuclear pyknosis (33.9‐fold higher than CTL and 2.1‐fold higher than SIVn), decreased molecular layer thickness (19.2% lower than CTL and 15.1% lower than SIVn), and reduced myelinated white matter area (31.5% lower than CTL and 25.3% lower than SIVn).

**FIGURE 1:**
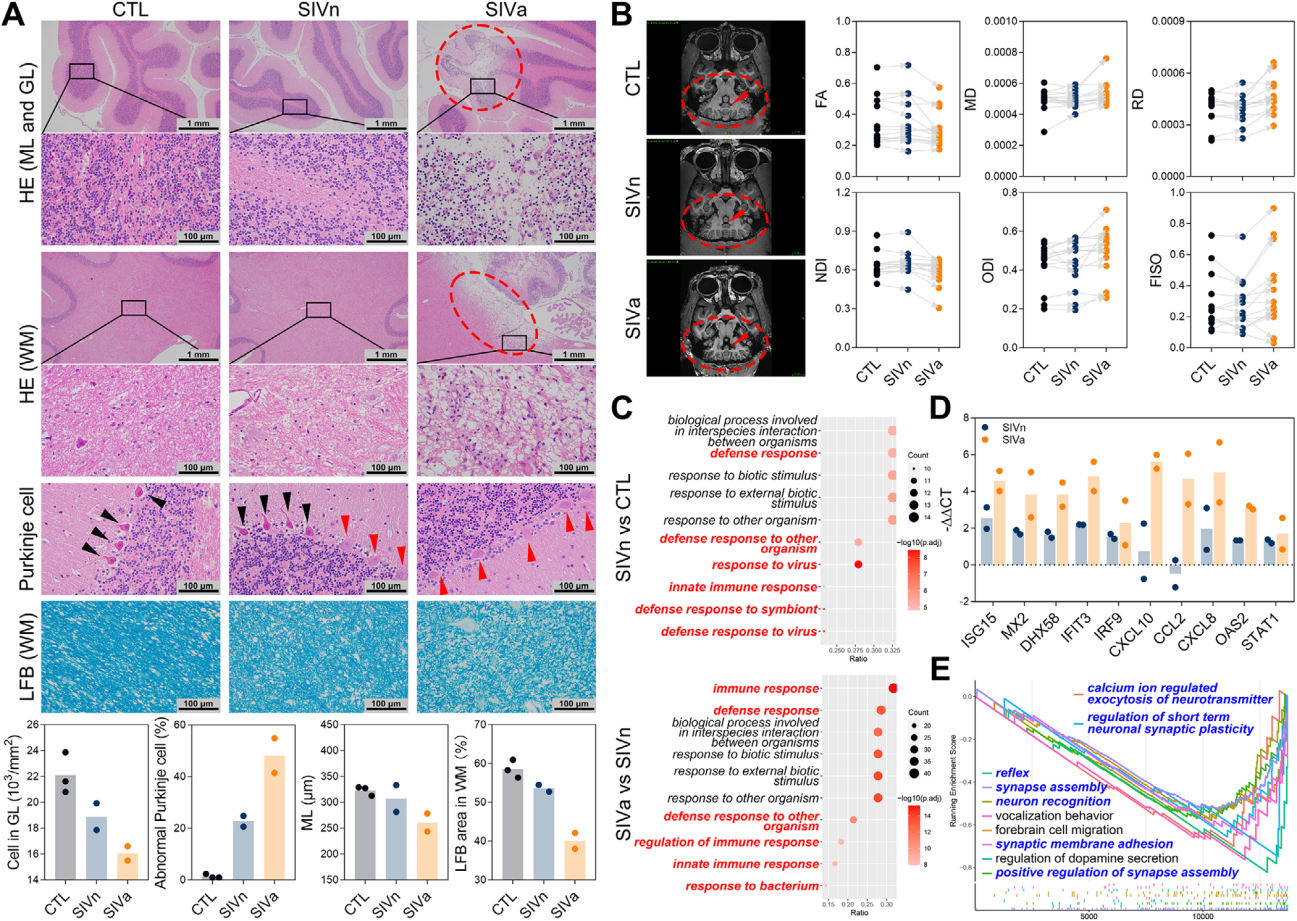
Histological, imaging, and transcriptomic analyses identify cerebellar alterations associated with ataxia. (A) Representative histological images and quantification showing severe cerebellar lesions in ataxic macaques (*n_CTL/SIVn/SIVa_* = 3/2/2). Each point represents the mean value from five randomly selected fields per animal. Red oval indicates structural disorganization. Black and red arrows indicate normal and degenerated Purkinje cells, respectively. H&E, hematoxylin and eosin; LFB, Luxol fast blue; ML, molecular layer; GL, granular layer; WM, white matter. (B) MRI showing cerebellar atrophy (red oval), fourth‐ventricle enlargement (red arrow), and altered neurite orientation dispersion and density imaging (NODDI) indices in ataxic macaques (*n_CTL/SIVn/SIVa_* = 1/1/1). Each point represents one cerebellar subregion defined using the D99 template. FA, fractional anisotropy; FISO, fraction of isotropic water; MD, mean diffusivity; NDI, neurite density index; ODI, orientation dispersion index; RD, radial diffusivity. (C) Over‐representation analysis (ORA) of the top 10 upregulated Gene Ontology Biological Process (GO‐BP) terms in the SIVn versus CTL comparison (top) and SIVa versus SIVn comparison (bottom), showing progressive enrichment of immune‐response pathways from CTL to SIVn to SIVa (*n_CTL/SIVn/SIVa_* = 3/2/2; terms ranked by adjusted *p* value). Red labels indicate immune‐ and defense‐related pathways. (D) Cerebellar mRNA expression of immune‐related genes (*n_CTL/SIVn/SIVa_* = 3/2/2), normalized to *RPL13A* and expressed relative to the CTL group. (E) Gene set enrichment analysis (GSEA) of the top 10 downregulated GO‐BP terms in the SIVa versus SIVn comparison, showing inhibition of neuro‐related pathways in the ataxic cerebellum (*n_SIVn_/_SIVa_* = 2/2; adjusted *p* < 0.05; terms ranked by normalized enrichment score [NES]). Blue labels indicate neuro‐related pathways.

MRI further confirmed macroscopic and microstructural cerebellar injury in ataxic macaques (Figure 1B). T1‑weighted imaging showed pronounced cerebellar atrophy and enlargement of the fourth ventricle in SIVa animals. Neurite orientation dispersion and density imaging (NODDI) across 14 cerebellar subregions further demonstrated widespread microstructural damage in SIVa macaques (Figure 1B), characterized by reduced fractional anisotropy (FA) and neurite density index (NDI), alongside elevated mean diffusivity (MD), radial diffusivity (RD), orientation dispersion index (ODI), and fraction of isotropic water (FISO). These abnormalities are consistent with imaging signatures of long‐term HIV‐1 infection, demyelination, neuroinflammation or edema, axonal disorganization, and neurite loss, collectively indicating severe tissue injury in the ataxic cerebellum [21, 22, 23, 24].

Bulk RNA sequencing (RNA‐seq) revealed a pronounced inflammatory signature in the ataxic cerebellum, together with suppression of neuronal signaling programs. Immune and defense‐related pathways were significantly enriched across all comparisons among CTL, SIVn, and SIVa animals (Figure 1C; Figure S1A), although the magnitude and breadth of this response were greatest in SIVa. ImmPort‐based annotation of upregulated immune‐related genes identified 89 differentially expressed genes (DEGs) in SIVa versus CTL and 51 DEGs in SIVa versus SIVn, compared with only 11 DEGs in SIVn versus CTL, indicating that the inflammatory program was most expanded in ataxic animals. Integration of the three comparisons into a merged protein–protein interaction (PPI) network identified antiviral response genes as the dominant hub module (Figure S1B), a transcriptional pattern validated by quantitative PCR (Figure 1D).

To assess inflammatory response specificity, cytokine levels were measured in plasma, cerebrospinal fluid (CSF), and cerebellar tissue homogenates (Figure S1C). In the SIVa group, significant cytokine elevations were confined to CSF (IP‐10, IL‐10, IL‐6, and IL‐8) and cerebellar homogenates (IP‐10, IFN‐α, and IL‐6), whereas plasma cytokine levels were not significantly increased, suggesting a nervous system‐enriched inflammatory response in ataxic animals. Given that relatively few downregulated DEGs were detected, gene set enrichment analysis (GSEA) was used to evaluate coordinated pathway‐level changes. GSEA revealed no significant suppression of neuronal pathways in the SIVn versus CTL comparison (Figure S1D, right), whereas multiple neuro‐related pathways were negatively enriched in both the SIVa versus CTL and SIVa versus SIVn comparisons (Figure S1D, left; Figure 1E). These findings indicate that neuronal pathway dysfunction is predominantly associated with the ataxic phenotype.

### 2.2 Specific SV40 Infection in the Ataxic Cerebellum

To define the cause of cerebellar injury in ataxic macaques, known etiologies of AIDS‐associated ataxia were systematically evaluated. Periodic acid‐Schiff (PAS) and Gram staining showed no evidence of bacterial or fungal infection in the cerebellum of SIVa animals (Figure 2A; Figure S2A,B). Dynamic T1‐weighted MRI sequences acquired at 5 frames/s did not reveal space‐occupying lesions, including lesions compatible with toxoplasmosis or tumor formation (Videos S3–S5). Vitamin B1 deficiency, a recognized cause of neurological dysfunction and ataxia in PLWH through impaired neurotransmitter synthesis and mitochondrial metabolism [25, 26], was also excluded (Figure 2B).

**FIGURE 2:**
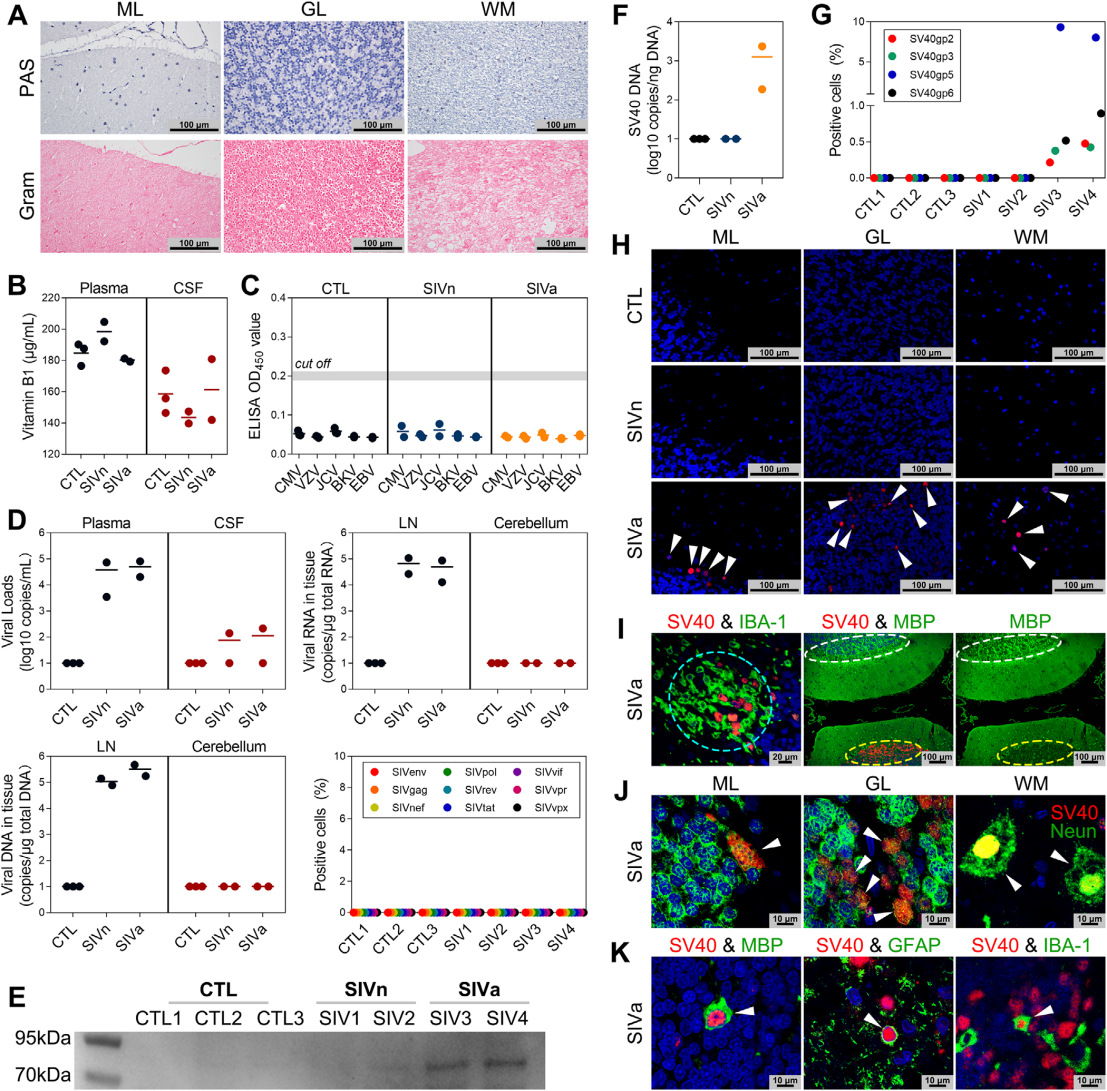
Etiological screening identifies SV40 infection as a feature of the ataxic cerebellum. (A) Representative histological images showing no evidence of bacterial or fungal infection in the SIVa cerebellum (*n_SIVa_* = 2). PAS, periodic acid‐Schiff. (B) Vitamin B1 levels in plasma and cerebrospinal fluid (CSF) across the three groups (*n_CTL/SIVn/SIVa_* = 3/2/2). (C) Viral antigen screening showing no evidence of cerebellar co‐infection with CMV, JCV, EBV, BKV, or VZV in ataxic macaques (*n_CTL/SIVn/SIVa_* = 3/2/2). BKV, BK virus; CMV, cytomegalovirus; EBV, Epstein‐Barr virus; JCV, John Cunningham virus; VZV, varicella‐zoster virus. (D) Assessment of systemic and tissue SIV burden showing comparable SIV infection status between SIVn and SIVa groups and no detectable cerebellar SIV infection (*n_CTL/SIVn/SIVa_* = 3/2/2). Top left, viral loads in body fluids; top right, SIV RNA in tissues; bottom left, SIV proviral DNA in tissues; bottom right, SIV RNA in snRNA‐seq data. LN, lymph node. (E−H) Detection of SV40 infection in the ataxic cerebellum (*n_CTL/SIVn/SIVa_* = 3/2/2). SV40 was assessed by detection of T antigen in CSF using western blotting (E), quantification of SV40 DNA in cerebellum by PCR (F), detection of viral RNA in snRNA‐seq data (G), and immunohistochemical localization of T antigen‐positive cells (white arrows) in cerebellar tissue (H). (I−K) Multiplex immunohistochemistry showing that SV40‐positive cells co‐localize with cerebellar lesions and predominantly involve granule cells (*n_CTL/SIVn/SIVa_* = 3/2/2). (I) Cyan oval indicates SV40‐positive cells surrounded by IBA‐1^+^ microglia. White and yellow ovals indicate MBP expression levels in SV40‐negative and SV40‐positive areas, respectively. (J) White arrows indicate SV40^+^NeuN^+^ cells. (K) White arrows indicate SV40^+^MBP^+^ cells (left), SV40^+^GFAP^+^ cells (middle), and SV40^+^IBA‐1^+^ cells (right).

The strong antiviral transcriptional signature in SIVa cerebellum prompted targeted screening for neurotropic viral pathogens. Antigens of cytomegalovirus (CMV), JCV, Epstein–Barr virus (EBV), BK virus (BKV), and varicella‐zoster virus (VZV) were not detected in cerebellar homogenates (Figure 2C). Systemic and lymphoid SIV burden also did not explain the ataxic phenotype. Plasma and CSF SIVmac239 viral loads, as well as proviral DNA and viral RNA levels in lymph nodes, showed no marked differences between SIVn and SIVa groups (Figure 2D). Moreover, SIV RNA and DNA were undetectable in cerebellar tissues from all infected macaques (Figure 2D), indicating an absence of productive SIV infection within the cerebellum.

SV40 showed a distinct disease‐associated pattern. SV40 T antigen in CSF and SV40 DNA and RNA in cerebellar tissue were detected exclusively in SIVa macaques (Figure 2E–G). Immunostaining further localized SV40^+^ cells to the granular layer, white matter, and molecular‑granular junction only in SIVa cerebellum (Figure 2H). These SV40^+^ foci were surrounded by IBA‑1^+^ microglia organized into nodular aggregates and exhibited reduced myelin basic protein (MBP) expression, linking local SV40 infection to focal microglial inflammation and demyelinating injury (Figure 2I; Figure S2C). Cell‐type co‐staining demonstrated that SV40 infection occurred primarily in NeuN^+^ neurons, whereas infection of IBA‐1^+^ microglia, GFAP^+^ astrocytes, and MBP^+^ oligodendrocytes was minimal (Figure 2J,K). These findings identify SV40 as a cerebellum‐specific viral pathogen in SIV‐infected macaques with ataxia and link neuronal SV40 infection to the major pathological features of the ataxic cerebellum, including microglial nodule formation and focal demyelination.

To determine whether SV40 infection reflected preexisting viral carriage and whether reactivation was associated with SIV‐related immune dysfunction, pre‐infection sera were examined for SV40 antibodies. All animals in the CTL, SIVn, and SIVa groups were SV40‐seropositive before SIV inoculation, consistent with the high seroprevalence reported in Chinese‐origin rhesus macaques (Figure S3A). Notably, SV40 antibody titers in SIVa animals declined substantially from pre‑infection to pre‑necropsy, whereas SIVn animals showed no such decline (Figure S3A), suggesting that SIV‑associated immune dysregulation may compromise control of SV40. Peripheral immune profiling further showed that, despite a shared SIV infection background, SIVa animals had more pronounced T‐cell activation and exhaustion than SIVn animals, with increased HLA‐DR^+^ and PD‐1^+^ CD4^+^ and CD8^+^ T‐cell populations (Figure S3B). These findings suggest that SV40 exposure was common across the cohort, whereas greater SIV‐associated immune dysregulation in ataxic animals may have created a permissive state for SV40 reactivation.

### 2.3 Predominant Granule Cell and Microglial Abnormalities in the Ataxic Cerebellum

To define the cellular alterations associated with ataxia, single‐nucleus RNA‐seq (snRNA‐seq) was performed on cerebellar samples from CTL, SIVn, and SIVa animals. After stringent quality control, 60 558 high‐quality nuclei were retained, with a median of 4,993 unique molecular identifiers (UMIs) and 2,191 genes detected per nucleus. Uniform manifold approximation and projection (UMAP) analysis showed well‐resolved cellular clustering after batch correction (Figure 3A; Figure S4A,B). Cell identities were assigned using canonical markers and established cerebellar reference datasets [27], yielding 11 major cell types (Figure 3A–C; Figure S4C). These included five neuronal populations, comprising GCs (GABRA6^+^), unipolar brush cells (UBCs; LMX1A^+^EOMES^+^), molecular layer interneurons (MLIs; LYPD6^+^SORCS3^+^), Golgi cells (LGI2^+^SLC6A5^+^), and Purkinje neurons (PNs; CA8^+^CALB1^+^), as well as six non‐neuronal populations, comprising Bergmann glia (BGs; FABP7^+^ADGRV1^+^), astrocytes (Astros; AQP4^+^ALDH1A1^+^), microglia (Micros; ENTPD1^+^P2RY12^+^), oligodendrocyte progenitor cells (OPCs; OLIG2^+^PCDH15^+^), oligodendrocytes (Oligos; MOG^+^FOLH1^+^), and vascular cells (Vass; DCN^+^C7^+^). In accordance with the cellular organization of the cerebellum, GCs represented the dominant nuclear population (Figure S4D, E). Consistent with findings in mouse cerebellum [27], two MLI subtypes marked by *MARCHF11* expression were resolved in macaques, with *NXPH1* expression restricted to MLI2.

**FIGURE 3:**
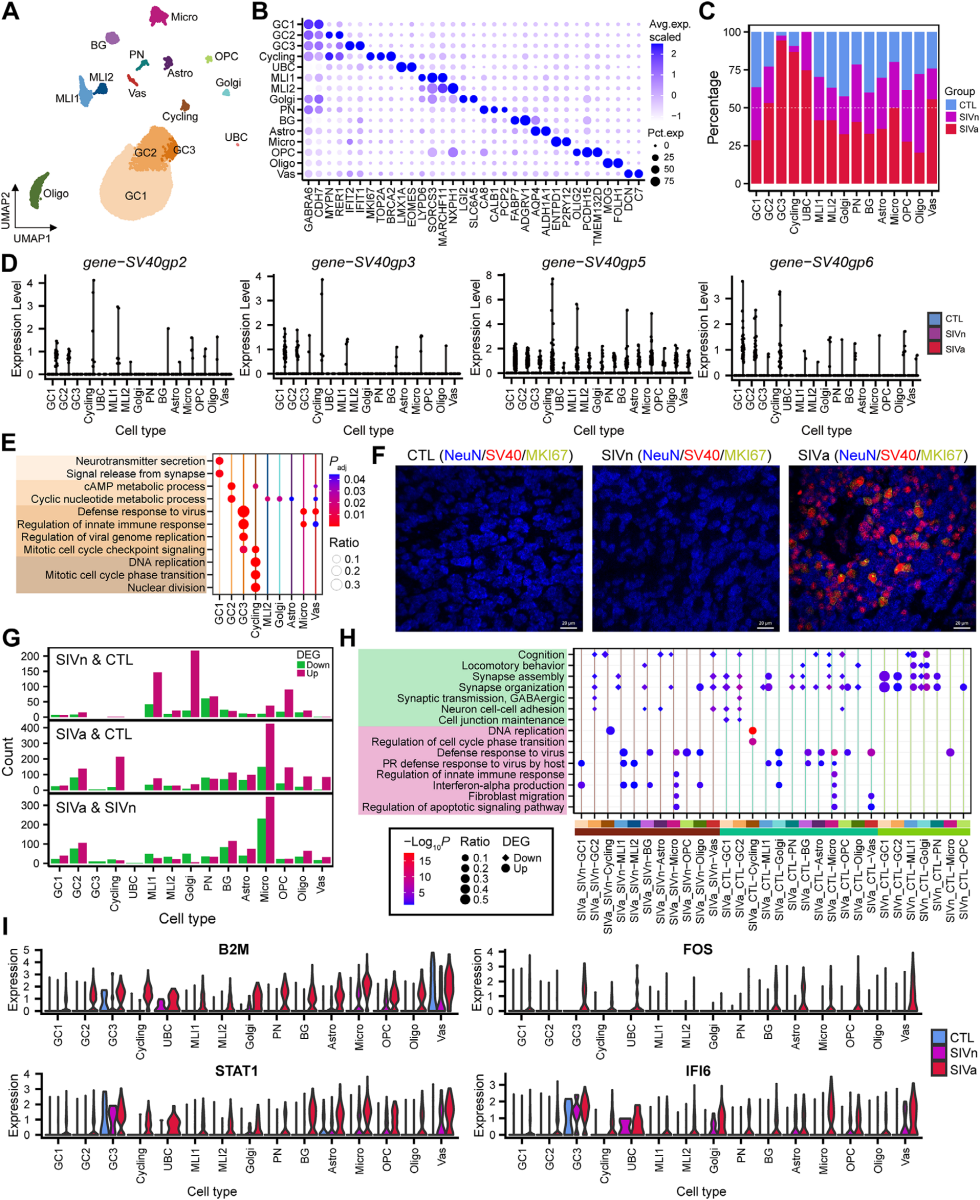
SnRNA‐seq identifies prominent granule cell and microglial transcriptional alterations in the ataxic cerebellum. (A) UMAP visualization of 60 558 high‐quality nuclei, color‐coded by annotated cell population. Astro, astrocyte; BG, Bergmann glia; Cycling, proliferating GC; GC, granule cell; Micro, microglia; MLI, molecular layer interneuron; OPC, oligodendrocyte progenitor cell; PN, Purkinje neuron; Oligo, oligodendrocyte; UBC, unipolar brush cell; Vas, vascular cell. (B) Dot plot showing marker gene expression across annotated cell populations. Dot size indicates percentage of nuclei expressing each gene (pct.exp) and color indicates scaled average expression (avg.exp.scaled). (C) Compositional analysis showing the relative abundance of each cell population across groups. (D) Violin plot showing SV40 viral gene expression restricted to SIVa samples. (E) Functional enrichment analysis of GC subtypes, including GC1, GC2, GC3, and cycling GCs. Dot size indicates gene ratio and color indicates adjusted *p* value. (F) Immunofluorescence validation of cerebellar sections stained for NeuN, SV40 T antigen, and proliferation marker MKI67. (G) Bar plot summarizing DEGs across cell types and pairwise comparisons (SIVa versus SIVn, SIVa versus CTL, and SIVn versus CTL). (H) Pathway enrichment analysis of DEGs. Rhombi and circles indicate downregulated and upregulated terms, respectively. Symbol size indicates gene ratio and color indicates −log10(adjusted *p* value). (I) Violin plots showing representative DEG expression patterns across groups.

SV40 transcripts were detected exclusively in SIVa samples and were concentrated predominantly in GCs (Figure 3D). Subclustering resolved four GC subpopulations: GC1 (CDH7^+^), GC2 (MYPN^+^RER1^+^), GC3 (IFIT1^+^IFIT2^+^), and a cycling population (MKI67^+^TOP2A^+^) (Figure S4F). Functional enrichment distinguished these states, with GC1 associated with synaptic signaling, GC2 with cAMP metabolism, GC3 with antiviral defense and mitotic checkpoint pathways, and cycling GCs with cell proliferation (Figure 3E). Relative to CTL and SIVn samples, GC2, GC3, and cycling GCs were enriched in SIVa cerebellum (Figure 3C; Figure S4G). Notably, proliferative NeuN^+^MKI67^+^ GCs were only found in SIVa cerebellum and co‐localized with SV40, linking viral infection to an aberrant GC state (Figure 3F).

CellChat analysis was then used to assess whether these cellular changes were accompanied by altered intercellular communication. Global interaction strength was markedly reduced in the ataxic cerebellum compared with SIVn and CTL cerebella (Figure S5A). Within the GC compartment, SIVa GC1 showed broad attenuation of both outgoing and incoming communication, whereas SIVa GC2 showed a more selective reduction in incoming signals (Figure S5B). In contrast, SIVa GC3, cycling GCs, and MLIs exhibited coordinated increases in both efferent and afferent signaling. These data indicate that the SV40‐positive ataxic cerebellum undergoes GC remodeling, characterized by expansion of GC2, antiviral GC3, and proliferative cycling GCs, together with a restructured communication network in which GC3, cycling GCs, and MLIs gain signaling influence while GC1 and GC2 lose connectivity.

Pairwise differential expression analysis identified extensive cell‐type‐specific transcriptional remodeling, with SIVa Micros showing the strongest perturbation (Figure 3G). Functional annotation revealed convergent disruption of neural programs and activation of antiviral inflammation (Figure 3H). Genes involved in cognition, locomotory behavior, and synapse assembly were downregulated across multiple SIVa cell populations, whereas genes required for cell junction maintenance were selectively reduced in SIVa GC1 and GC2, consistent with the weakened intercellular communication observed in these states. In parallel, antiviral response genes were broadly induced in SIVa cells, most prominently in Micros, and apoptosis‐associated genes were selectively upregulated in SIVa Micros and Vass. In particular, four representative DEGs captured distinct components of this pathological program (Figure 3I). *B2M*, which encodes β2‐microglobulin, was broadly upregulated in SIVa cells and has been linked to amyloid pathology and cognitive decline in Alzheimer's disease [28]. *FOS* was markedly increased in neuronal populations, including GC3, cycling GCs, and UBCs, as well as in Astros and Micros. Given its roles in proliferation, differentiation, cellular transformation, neuronal depolarization, and glial inflammatory responses [29, 30], this pattern indicates coordinated neuronal and glial activation. Immune mediators *STAT1* and *IFI6* were also consistently upregulated, indicating sustained interferon signaling and antiviral defense. Collectively, these transcriptional changes support a model in which SV40‐associated expansion of GC2, GC3, and cycling GCs is linked to cerebellar circuit disruption, while microglial hyperactivation amplifies local neuroinflammation and contributes to ataxia pathogenesis.

### 2.4 SV40 Infection Triggers Neuronal Pyroptosis and Proinflammatory Signaling

To determine how SV40 affects neural cells directly, an in vitro infection model was established in SH‑SY5Y human neuroblastoma cells. SV40 productively infected SH‑SY5Y cells, as shown by dose‐dependent increases in viral DNA in both cell lysates and culture supernatants, indicating efficient viral entry, replication, and release (Figure 4A).

**FIGURE 4:**
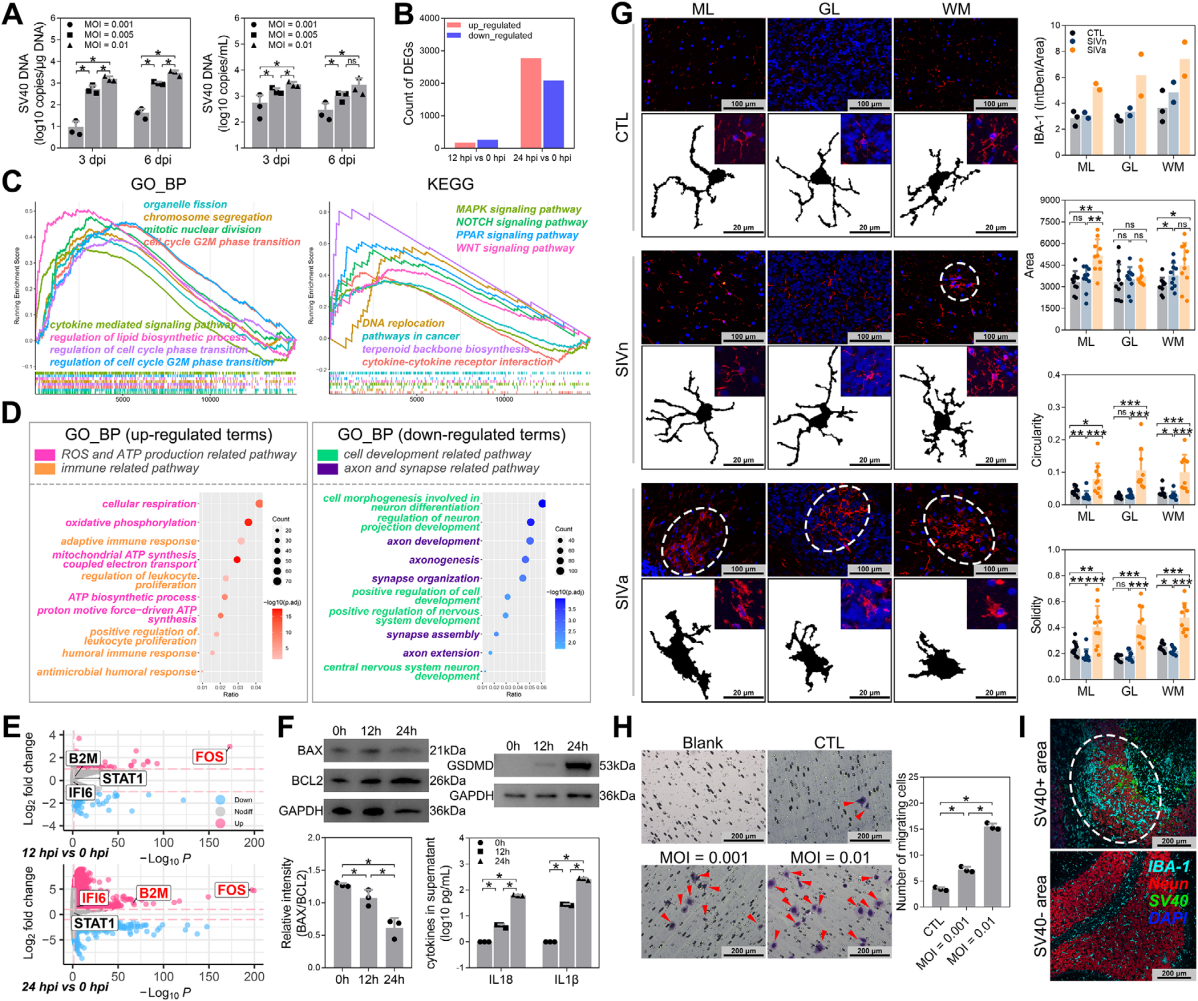
In vitro SV40 infection triggers neuronal injury and promotes downstream microglial activation and recruitment. (A) SV40 effectively infects SH‐SY5Y cells. Viral DNA was quantified in cell lysates (left) and culture supernatants (right) after infection at multiplicities of infection (MOIs) of 0.01, 0.005, or 0.001, and assessment at 0, 3, and 6 days post‐infection (dpi) (*n* = 3 per condition). (B) Number of DEGs in SH‐SY5Y cells infected with SV40 at an MOI of 0.01, comparing 12 and 24 h post‐infection (hpi) with 0 hpi (*n* = 3 per condition). (C) Gene set enrichment analysis (GSEA) showing significant enrichment of cell‐cycle‐related pathways in SV40‐infected SH‐SY5Y cells at 12 hpi versus 0 hpi (adjusted *p* < 0.05; *n* = 3 per condition). (D) Over‐representation analysis (ORA) showing pathway dysregulation in SV40‐infected SH‐SY5Y cells at 24 hpi versus 0 hpi. Upregulated pathways included reactive oxygen species (ROS) production, ATP metabolism, and immune responses, whereas downregulated pathways were related to cell development and axon/synapse function (adjusted *p* < 0.05; *n* = 3 per condition). (E) Volcano plot showing expression changes in *FOS*, *B2M*, *STAT1*, and *IFI6* in SV40‐infected SH‐SY5Y cells at 12 hpi versus 0 hpi (top) and 24 hpi versus 0 hpi (bottom) (*n* = 3 per condition). (F) SV40 infection induces pyroptotic, rather than apoptotic, injury in SH‐SY5Y cells. Pyroptosis was assessed by GSDMD expression using western blotting and by IL‐1β and IL‐18 levels using ELISA. Apoptosis was assessed using the BAX/BCL2 ratio by western blotting (*n* = 3 per condition; MOI = 0.01). (G) IBA‐1 staining showing microglial activation, nodule formation (white oval), and morphological changes in the ataxic cerebellum (*n_CTL/SIVn/SIVa_* = 3/2/2). Quantification confirmed increased IBA‐1^+^ mean fluorescence intensity, with each point representing the mean value of five randomly selected fields per animal, and altered morphometric parameters, including area, circularity, and solidity, calculated from 10 randomly selected microglia per group. (H) Conditioned medium from SV40‐infected SH‐SY5Y cells at 12 hpi promotes HMC3 migration (red arrow) in a Transwell assay (*n* = 3). (I) Within‐animal comparison in SIV3 showing that SV40‐infected granule neurons (SV40^+^/NeuN^+^) are associated with microglial nodule formation (IBA‐1^+^; white oval) in infected regions but not in adjacent SV40‐negative cerebellar regions. ns: not significant; *p < 0.05; **p < 0.01; ***p < 0.001.

The SV40 replication cycle comprises two major phases [31]. During the early phase, the viral chromatin core is delivered to the nucleus and initiates early transcription, leading to large T antigen and small t antigen expression at approximately 12 h post‐infection (hpi). During the late phase, viral DNA replication, late transcription, and synthesis of the capsid proteins VP1, VP2, and VP3 drive virion assembly, followed by viral release and cytopathic injury at approximately 24 hpi. Based on these established kinetics, SH‐SY5Y cells infected at a multiplicity of infection (MOI) of 0.01 were harvested at 0, 12, and 24 hpi for RNA‐seq analysis (Figure 4B). At 12 hpi, corresponding to the phase of T/t antigen production, cell‐cycle transition and proliferation pathways were selectively activated (Figure 4C), supporting the interpretation that the proliferative cycling GC phenotype observed in vivo originated from SV40 infection. By 24 hpi, when viral release and cytopathic effects were evident, SV40 infection induced a broader injury program in SH‐SY5Y cells (Figure 4D), characterized by enrichment of pathways related to reactive oxygen species (ROS) production, ATP metabolism, and immune responses, together with suppression of cell‐development and axon‐development programs, suggesting a shift from early proliferative activation to cellular injury and impaired neuronal function. To assess whether the in vitro model reproduced the transcriptional features of the ataxic cerebellum, four representative DEGs identified in SIVa cerebellum were examined (Figure 3I). Three were significantly upregulated in SV40‑infected SH‑SY5Y cells at 24 hpi (Figure 4E), supporting a conserved SV40‑associated pathological signature across the cellular model and in vivo cerebellar tissue.

The fate of SV40‐infected SH‐SY5Y cells was further assessed using key markers of apoptosis (BAX/BCL2 ratio) and pyroptosis (GSDMD, IL‐1β, IL‐18). The BAX/BCL2 ratio declined progressively after infection relative to 0 hpi (Figure 4F), consistent with the established anti‐apoptotic function of SV40 T/t antigen [32]. In contrast, GSDMD, IL‐1β, and IL‐18 protein levels increased markedly, particularly at 24 hpi (Figure 4F), identifying pyroptosis as a dominant mechanism of SV40‐induced neuronal injury. Pyroptosis is a lytic form of programmed cell death mediated by gasdermin pore formation, plasma membrane rupture, and release of proinflammatory mediators that recruit immune cells [33]. Consistent with this mechanism, SV40 infection induced robust secretion of IP‐10, IL‐6, TNF‐α, IFN‐α/β/γ, and IL‐12 into the culture supernatant at 24 hpi (Figure S6). These findings suggest that SV40‐infected neurons act as a primary trigger of neuroinflammation in the ataxic cerebellum, thereby promoting the recruitment and activation of immune cells and the formation of nodular aggregates.

### 2.5 Microglial Recruitment and Nodule Formation Amplify SV40‐Induced Pathology

To identify the immune compartment associated with cerebellar inflammation, infiltrating immune cells (CD3^+^ T cells and IBA‑1^−^CD163^+^ macrophages) and resident glial populations (microglia and astrocytes) were examined. CD3^+^ T‐cell and macrophage infiltration did not differ significantly in SIVa cerebellum compared with CTL and SIVn cerebella (Figure S7A,B). Astrocyte reactivity, assessed by GFAP signal intensity, was also comparable between SIV‑infected groups (Figure S7C,D), suggesting that neither peripheral immune‐cell infiltration nor astrocytic activation accounted for the ataxia‐associated inflammatory phenotype.

In contrast, microglia showed prominent activation in the ataxic cerebellum. IBA‑1^+^ signal intensity was markedly increased in SIVa tissue, and microglial nodules were distributed across the molecular, granular, and white‑matter layers (Figure 4G). This pattern was observed only sporadically in SIVn tissue, indicating selective microglial recruitment and aggregation in ataxic animals. Morphometric analysis further showed increased circularity and solidity in SIVa microglia, consistent with a transition from ramified surveillant morphology to an ameboid reactive state. Microglia detect viral pathogen‐associated molecular patterns (PAMPs) through pattern‐recognition receptors (PRRs), enabling them to mount early innate immune responses during CNS viral infection [34, 35]. In neurotropic viral infections such as coxsackievirus B3 and herpes simplex virus type 1 (HSV‐1), neurons can support robust viral replication with limited IFN responses, whereas microglia mount strong antiviral activation and serve as a principal source of type I IFN in the CNS [36, 37]. The microglial response to SV40 was therefore examined in vitro. In contrast to the progressive transcriptional changes observed in SH‐SY5Y neurons, HMC3 microglial cells mounted rapid immune responses to SV40 at 12 and 24 hpi, with significant enrichment of immune‐ and defense‐related pathways and increased expression of antiviral genes, including *ISG15*, *STAT1*, *OASL*, and *IFIT3* (Figure S8). Conditioned medium from SV40‑infected SH‑SY5Y neurons strongly promoted HMC3 migration in Transwell assays (Figure 4H). Consistent with this chemotactic response, SIVa cerebellum showed dense microglial accumulation around SV40‐infected neurons within SV40‐positive regions (Figure 4I), culminating in microglial nodule formation.

Together, these findings indicate that SV40‐infected cerebellar neurons initiate pyroptotic injury and inflammatory cytokine release, which recruit and activate microglia. The resulting microglial response amplifies local neuroinflammation and drives the formation of nodular aggregates, identifying microglia as central effectors of SV40‑induced cerebellar pathology.

## 3 Discussion

This study identified cerebellar SV40 infection as a direct viral cause of neurodegeneration and neuroinflammation in chronically SIV‐infected rhesus macaques with ataxia. Integration of histopathology, MRI neuroimaging, bulk transcriptomics, single‐nucleus transcriptomics, and in vitro infection assays demonstrated that SV40 infection triggered pyroptosis and inflammatory cytokine release in cerebellar granule neurons. This neuronal injury program promoted microglial recruitment and activation, intensified local neuroinflammation, and culminated in nodular aggregate formation. The absence of cART exposure in these chronically SIV‐infected macaques enabled the model to recapitulate key features of cerebellar lesion pathogenesis in untreated HIV infection while allowing viral mechanisms of AIDS‐associated ataxia to be examined without the confounding influence of antiretroviral neurotoxicity.

Given the scarcity of autopsy material from PLWH with ataxia, the cerebellar pathology of this disorder has remained largely descriptive, inferred mainly from T1‐weighted abnormalities and histological findings of cerebellar atrophy, fourth‐ventricle enlargement, GC loss, axonal swelling, and white matter degeneration [8, 21]. Ataxic SIV‐infected macaques reproduced this full spectrum of neuroimaging and histopathological abnormalities, supporting close pathological correspondence with HIV‐associated ataxia in humans. Deeper analysis resolved the ataxic cerebellar phenotype into three linked pathological domains: macrostructural injury, including diffuse atrophy and focal tissue disorganization; cellular damage, including neuronal degeneration and demyelination; and neuroimmune remodeling, marked by microglial activation, migration, and glial nodule formation. Together, these convergent abnormalities provide an integrated framework for defining cerebellar injury in AIDS‐associated ataxia.

To determine whether the ataxic cerebellar lesions reflected SV40‐mediated neuropathogenesis rather than another recognized cause of AIDS‐associated ataxia, established contributors other than antiretroviral toxicity were systematically evaluated, including advanced AIDS, opportunistic infections, mass‐forming lesions, and nutritional deficiency. After alternative etiologies were excluded, SV40 remained the only etiological candidate selectively associated with the ataxic cerebellar phenotype. This association is biologically plausible as SV40 and JCV employ conserved early‐region programs during productive infection, in which large T antigen and small t antigen redirect host‐cell S‐phase machinery [22]. Large T antigen orchestrates viral DNA replication as an ATP‐dependent helicase dodecamer while hijacking pRb‐ and p53‐mediated cell‐cycle checkpoints, forcing S‐phase entry and limiting intrinsic apoptosis. Simultaneously, small t antigen reinforces this permissive state by inhibiting PP2A, activating proliferative signaling and attenuating extrinsic apoptosis through the Akt signaling pathway. Critically, the pathological consequence of this early‐region program is determined by cellular permissiveness. In permissive cells, including monkey kidney cells, SV40 induces lytic cytopathic injury marked by vacuolation and lysosomal enzyme release [23, 24]. In non‐permissive cells, such as human mesothelial cells and monkey astrocytes, sustained T antigen activity instead promotes oncogenic transformation through pRb and p53 inactivation [25, 26]. JCV shows a comparable context‐dependent pattern in the nervous system, causing lytic demyelination in oligodendrocytes while also promoting glial tumorigenesis, including glioblastoma and medulloblastoma, through large T antigen‐driven cell–cycle dysregulation and small t antigen‐mediated inhibition of apoptosis [38]. In PLWH, cerebellar JCV infection causes enlargement of infected GCs, followed by scattered destructive foci within the granular layer [39]. The same precise pathological sequence in SV40‐infected primate cerebellum suggests that SV40 produces a JCV‐like pattern of GC infection, cytopathic injury, and focal cerebellar destruction [40].

In the ataxic cerebellum, SV40 was predominantly localized to NeuN^+^ cerebellar GCs, as confirmed by co‐staining for SV40 T antigen. This neuronal tropism was accompanied by a selective reorganization of the GC compartment. SnRNA‐seq identified three disease‐relevant alterations within the GC compartment: reduced bidirectional signaling in the GC1 subpopulation, emergence of a proliferative cycling GC subpopulation, and antiviral activation in the GC3 subpopulation. The functional significance of impaired GC communication is supported by previous research showing that the elimination of CaV2.1, CaV2.2, and CaV2.3 channels from GCs is sufficient to produce ataxia‐like motor deficits [41]. Given that GC1 constituted the dominant GC population, loss of GC1‐centered communication in SIVa cerebellum likely represents a major contributor to impaired motor coordination. CellChat analysis further indicated that cerebellar communication was remodeled differently in SIVa and SIVn animals. SIVa showed more inferred interactions but lower global interaction strength, whereas SIVn showed increases in both interaction number and interaction strength compared with CTL. In SIVa, the expanded participation of GC3, cycling GCs, and MLIs may have increased the number of inferred interactions, but this shift did not offset the pronounced loss of both outgoing and incoming GC1 signaling or the reduction in incoming GC2 signaling. As GC1 contributed substantially to the overall communication network, GC1 impairment likely drove the reduction in global interaction strength, despite the larger number of inferred interactions. In contrast, SIVn animals showed enhanced signaling across multiple cell types, particularly GC1, GC2, and oligodendrocytes, with increased bidirectional GC1 signaling likely accounting for the concurrent rise in interaction number and strength.

Virus‐induced neuronal pyroptosis is increasingly recognized as a central pathogenic mechanism of neurological injury in infections caused by HSV‐1, enterovirus 71, Zika virus, West Nile virus, and rabies virus [42, 43, 44, 45, 46]. SV40 infection in SH‐SY5Y cells extended virus‐induced neuronal pyroptosis to polyomavirus‐associated neurotoxicity, with productive infection inducing GSDMD‐associated pyroptosis, IL‐1β and IL‐18 release, and a broader proinflammatory cytokine response. Unlike caspase‐3/7‐dependent apoptosis, which generally preserves plasma membrane integrity, pyroptosis disrupts the membrane through gasdermin pore formation downstream of inflammatory caspases, including caspase‐1, ‐4, ‐5, and ‐11, and releases proinflammatory cytokines that recruit and activate immune cells [47]. Consistent with this mechanism, SV40‐infected SH‐SY5Y cells released proinflammatory mediators that promoted microglial migration and activation, while SV40‐positive neuronal foci in the ataxic cerebellum were surrounded by activated microglial aggregates that formed nodules. Given that proinflammatory microglial activation can trigger cerebellar ataxia through Purkinje cell hyperexcitation [48], these findings indicate that SV40‐induced neuronal injury drives downstream microglial activation, thereby exacerbating cerebellar dysfunction and promoting ataxic pathology.

All enrolled animals were SV40‑seropositive prior to SIV inoculation, consistent with prior SV40 exposure and established viral latency before SIV infection. In SIVa animals, the marked reduction in SV40 antibody titers occurred together with stronger T‑cell activation and exhaustion, suggesting weakened immune containment of latent SV40. This temporal relationship is more consistent with severe SIV‑associated immune deterioration permitting SV40 reactivation than with SV40 reactivation as the initiating event. Reactivated SV40 may then further compromise antiviral immunity, as SV40 can downregulate CD83 and CD86 on dendritic cells and impair T‑cell activation [49], while SV40‐encoded microRNAs reduce susceptibility to cytotoxic T‑cell‐mediated lysis [50]. These interactions support a reciprocal process in which immune dysfunction enables SV40 reactivation, and SV40 reactivation further reinforces local and systemic immune dysregulation. Here, both macaques that developed ataxia were female, raising the possibility of sex‐related vulnerability. However, evidence for sex effects in HIV‐associated neurological disease remains inconsistent even for HAND [51] and is far more limited for rare movement disorders. In a previous systematic review, pooled case‐series data revealed numerical male predominance in myoclonus (30 of 32 cases), chorea (19 of 26 cases), and ataxia (100 of 148 cases), whereas opsoclonus‐myoclonus‐ataxia syndrome showed numerical female predominance (10 of 17 cases) [2]. These estimates were derived from aggregated case reports rather than population‐based cohorts. Therefore, despite the occurrence of ataxia in two female animals in this study, the sample size (*n* = 2 per infected group) precludes any meaningful inference regarding sex predisposition to SV40‐induced cerebellar pathology.

This study has several limitations. First, the low natural incidence of ataxia in SIV‐infected rhesus macaques restricted the symptomatic group to two animals. Although both ataxic macaques showed consistent etiological, histopathological, and transcriptomic profiles, the small sample size limits statistical inference, restricts assessment of interindividual variation, and precludes determination of whether SV40 infection consistently produces ataxia across infected animals. Therefore, larger cohorts are needed to establish reproducibility and define the range of SV40‐associated cerebellar disease. Second, direct intracranial SV40 inoculation in SIV‐infected rhesus macaques would provide a more definitive approach for testing neuropathogenic mechanisms in an immunosuppressed host. However, the generation of such a dual‐pathogen model is prohibited under current biosafety regulations in China. Third, although serological findings support preexisting SV40 exposure followed by reactivation, the cohort was too small to identify host‐specific factors that determine why only some SIV‐infected macaques developed clinical ataxia. Fourth, proof‐of‐concept studies with SV40‐directed antivirals or inhibitors of pyroptosis also remain to be performed. Fifth, while mechanistic experiments were performed using human SH‐SY5Y neuroblastoma cells and HMC3 microglial cells as established models, these immortalized cell lines may not fully recapitulate the physiological properties of primary macaque cerebellar granule neurons and microglia. Sixth, because tissue collection focused on the cerebellum, potential SV40 involvement in other structures that contribute to ataxia, including the brainstem, thalamus, and vestibular system, was not assessed. Broader neuroanatomical sampling will be required to determine whether SV40 pathology extends beyond the cerebellum. Seventh, although the data support pyroptosis as a major inflammatory cell‐death pathway in SV40‐infected SH‐SY5Y cells, parallel screening of other inflammatory death programs, such as necroptosis, would provide a more comprehensive view of SV40‐induced neural injury.

In conclusion, this study identified SV40 as a direct etiological agent of cerebellar lesions in SIV‐infected macaques with ataxia and defined a pathogenic sequence involving neuronal infection, pyroptotic injury, and microglial activation. These findings establish a mechanistic model of polyomavirus‐driven cerebellar injury in immunodeficiency and provide experimental evidence that viral reactivation can contribute directly to AIDS‐related motor dysfunction. Therapeutic strategies that target polyomavirus replication or suppress pyroptosis‐associated neuroinflammation may therefore warrant investigation for ataxia in PLWH.

## 4 Materials and Methods

### 4.1 Animals

Seven adult rhesus macaques were obtained from the Kunming Primate Research Center, Kunming Institute of Zoology (KIZ), Chinese Academy of Sciences (CAS). Animals were maintained under standard housing conditions at 24°C with a 12‐h light/dark cycle and ad libitum access to food and water. Macaques in the SIVn and SIVa groups were intravenously inoculated with 4000 50% tissue culture infectious doses (TCID_50_) of SIVmac239 (GenBank: M33262.1) and developed chronic infection, characterized by sustained viral persistence for more than 3 years without AIDS‐defining clinical manifestations such as cachexia or chronic diarrhea. No animals received cART during the study period.

### 4.2 Sample Collection

All procedures were performed under anesthesia induced by intramuscular injection of Zoletil 50 (Virbac, France). Peripheral blood and CSF were collected from the posterior tibial vein and by lumbar puncture, respectively. Following euthanasia, animals underwent transcardial perfusion with pre‐cooled phosphate‐buffered saline (PBS). Multiple tissues were then collected, including testes, colon, spleen, liver, lungs, lymph nodes, and cerebellum.

### 4.3 Histological and Immunological Staining

Cerebellar tissues were fixed in 4% paraformaldehyde, dehydrated through a graded ethanol series, embedded in paraffin, and sectioned at 4 µm. Histopathological changes were assessed using hematoxylin and eosin (H&E), Luxol fast blue (LFB), PAS, and Gram staining, followed by imaging with a brightfield microscope (BX53, Olympus, Japan). Single‐target immunofluorescence staining was performed according to standard protocols [52]. Multiplex immunohistochemical staining was performed using tyramide signal amplification (TSA), as described previously [53]. Detailed information on reagents and analytical procedures is provided in the Supporting Information Materials.

### 4.4 MRI Assay

Anesthesia was induced with ketamine HCl (ZMBK, China) and maintained with Dexdomitor (Zoetis, USA). Whole‐brain MRI was performed in rhesus macaques before euthanasia using a UMR 790 3T scanner (United Imaging, China). The imaging protocol included T1‐weighted and NODDI sequences. Preprocessed data were registered to the D99 macaque brain atlas for automated segmentation and regional brain‐volume extraction [54]. Diffusion and NODDI metrics were then processed using dtifit (https://fsl.fmrib.ox.ac.uk/fsl/docs/#/) and AMICO (https://www.github.com/daducci/AMICO). Fourteen regions in each cerebellar hemisphere were segmented for analysis. Detailed imaging parameters are provided in the Supporting Information Materials.

### 4.5 Cytokine Quantification by Luminex xMAP Assay

Cytokine concentrations in CSF, plasma, and supernatants from cerebellar tissue homogenates were quantified using an NHP XL Cytokine Luminex Performance Premixed Kit (FCSTM21, R&D Systems, USA) according to the protocol supplied by the manufacturer.

### 4.6 Flow Cytometry

Flow cytometry was performed as described previously [55]. Absolute T‐cell counts were determined using fresh EDTA‐K2‐anticoagulated peripheral blood. Data were acquired on an LSRFortessa Cell Analyzer (BD Biosciences, USA) and analyzed using FlowJo v10.5.3 software (FlowJo LLC, USA). Detailed antibody information is provided in the Supporting Information Materials.

### 4.7 SV40 Infection in Vitro

SH‐SY5Y human neuroblastoma cells and HMC3 human microglial cells were purchased from Servicebio (China). Mycoplasma contamination was monitored routinely throughout the study using PCR and microscopic examination. SH‐SY5Y cells (1 × 10^6^) were seeded in six‐well plates and cultured overnight before infection with SV40 for 1 h at MOIs of 0.01, 0.005, or 0.001. Cells were then washed twice with PBS to remove unbound viral particles, after which fresh MEM/F12 medium (PM151220A, Procell, China) supplemented with 10% fetal bovine serum (FBS) was added. For SV40 DNA quantification, culture supernatants and cells were collected at 3 and 6 days post‐infection (dpi) for all MOIs. For transcriptome sequencing, western blot analysis, and cytokine profiling, SH‐SY5Y cells and supernatants were collected at 0, 12, and 24 hpi after infection at an MOI of 0.01. HMC3 cells (4 × 10^5^) were seeded in six‐well plates in MEM/NEAA medium (PM150410, Procell, China) supplemented with 10% FBS. HMC3 cells infected with SV40 at an MOI of 0.01 were collected at 0, 12, and 24 hpi for transcriptome sequencing. All treatments were performed in triplicate.

### 4.8 PCR Assay

Total RNA was extracted from CSF and plasma using a High Pure Viral RNA Kit (11858882001, Roche, Switzerland), and from tissues and cell lines using TRIzol Reagent (9109, Takara, Japan). Genomic DNA was purified from tissues, SH‐SY5Y cells, and culture supernatants using a TIANamp Genomic DNA Kit (DP304, TIANGEN, China). RNA was reverse transcribed into cDNA using a PrimeScript FAST RT Reagent Kit with gDNA Eraser (RR092A, Takara, Japan). Nucleic acid concentrations were quantified with a NanoDrop ND2000 spectrophotometer (Thermo, USA).

SIVmac239 viral RNA and DNA were quantified using primer and probe sequences targeting the SIV *gag* gene on a QuantStudio 5 Real‐Time PCR Instrument (Thermo). Viral RNA was quantified using an Evo M‐MLV One‐Step RT‐qPCR Kit (AG11715, Accurate Biotechnology, China), whereas viral DNA was quantified using the Premix Ex Taq (RR390A, Takara). Standard curves were generated from serial dilutions of the pGEM‐4Z‐SIV gag357 plasmid. SV40 DNA in cerebellar tissues, SH‐SY5Y cells, and culture supernatants was detected using a Simian Virus 40 Probe qPCR Kit (YLK‐PCR0316, YLKbio, China) according to the protocol supplied by the manufacturer. Target‐gene cDNA was amplified using TB Green Premix Ex Taq II (RR820A, Takara), with *RPL13A* used as the internal reference gene. Relative gene expression was calculated as ΔCt = Ct (target gene) − Ct (*RPL13A*). Primer and probe sequences are listed in Table S2.

### 4.9 Western Blot Assay

CSF or supernatants from homogenized SH‐SY5Y cells were mixed with 5× loading buffer (P0286, Beyotime, China), separated on 4%–15% Tris‐Gly Precast Gels (BL1006A, Biosharp, China) or FuturePAGE 4%–12% Precast Gels (ET12412Gel, ACE, China), and transferred onto polyvinylidene difluoride (PVDF) membranes (1620177, Bio‐Rad, USA). Membranes were blocked with 5% skimmed milk for 2 h at room temperature, incubated with primary antibodies overnight at 4°C, and then incubated with corresponding horseradish peroxidase (HRP)‐conjugated secondary antibodies for 1 h at room temperature. All blots were developed using a chemiluminescent HRP substrate (WBKLS0500, Merck Millipore, USA) and analyzed using ImageJ software. Detailed antibody information is provided in the Supporting Information Materials.

### 4.10 Enzyme‐Linked Immunosorbent Assay (ELISA)

Commercial ELISA kits were used to quantify viral antigens, SV40 antibodies, cytokines, and vitamin B1. Detailed information is provided in the Supporting Information Materials.

### 4.11 Bulk and Single‐Nucleus RNA Sequencing

Bulk RNA‐seq and snRNA‐seq were conducted on cerebellar tissues from all animals and on SV40‐infected SH‐SY5Y and HMC3 cells. Total RNA or nuclei were isolated and used for library construction according to standard protocols. All libraries were sequenced on the Illumina NovaSeq 6000 platform. For bulk RNA‐seq, clean reads were aligned to species‐specific reference genomes (*Macaca mulatta* Mmul_10 or *Homo sapiens* GRCh38). DEGs were identified using DESeq2 and subjected to functional enrichment analysis. For snRNA‐seq, raw sequencing reads were processed to generate gene‐expression matrices, followed by stringent quality control, normalization, and dataset integration. Cell clusters were annotated using established marker genes. Downstream analyses included cell‐type‐specific differential expression, cell–cell communication inference, and pathway enrichment. Complete methodological details are provided in the Supporting Information Materials.

### 4.12 Transwell Migration Assay

HMC3 cells (2 × 10^4^) suspended in 100 µL of serum‐free MEM/NEAA medium were seeded into the upper chamber of 8.0‐µm‐pore 12‐well Transwell inserts (14242, LABSELECT, China). Conditioned medium from SH‐SY5Y cells incubated with or without SV40 (MOI = 0.01, 12 h) was mixed with fresh medium containing 10% FBS at a 2:3 ratio, and 500 µL of the mixture was added to the lower chamber. After incubation for 12 h at 37°C, migrated cells were fixed with 4% paraformaldehyde for 30 min and stained with 0.1% crystal violet (C0121, Beyotime, China). Non‐migrated cells were removed from the upper surface of the membrane. Migrated cells were counted under an inverted microscope (DMI4000B, Leica, Germany) at 20× magnification in five randomly selected fields per well, and results were expressed as the mean number of cells per field.

### 4.13 Statistical Analyses

Mann–Whitney tests were used to analyze MBP expression levels, SV40 DNA levels in cells and supernatants, microglial morphological indices, BAX/BCL2 ratios, cytokine levels in culture supernatants, and HMC3 migration. Statistical analyses were performed using GraphPad Prism v8.0.1 software (GraphPad Software, USA). Experimental data are presented as mean ± standard deviation (SD). *p* < 0.05 was considered statistically significant.

## Author Contributions

Conceptualization: Yong‐Tang Zheng, Dong‐Dong Wu, Tian‐Zhang Song, and Si‐Dong Xiong. Data curation: Tian‐Zhang Song, Li‐Rong Zhang, Yun‐Mei Wang, Yan Hu, Qing Li, Ren‐Rong Tian, and Liu‐Meng Yang. Data interpretation: Tian‐Zhang Song, Li‐Rong Zhang, and Yun‐Mei Wang. Formal analysis: Tian‐Zhang Song, Li‐Rong Zhang, and Yun‐Mei Wang. Investigation: Yong‐Tang Zheng, Dong‐Dong Wu, Si‐Dong Xiong, Tian‐Zhang Song, and Yun‐Mei Wang. Project administration: Yong‐Tang Zheng, Dong‐Dong Wu, and Tian‐Zhang Song. Software: Tian‐Zhang Song, Li‐Rong Zhang, and Yun‐Mei Wang. Supervision: Yong‐Tang Zheng and Dong‐Dong Wu. Writing: Yong‐Tang Zheng, Tian‐Zhang Song, Li‐Rong Zhang, and Yun‐Mei Wang. All authors have read and approved the final manuscript.

## Funding

This study was supported by grants from the National Natural Science Foundation of China (U23A20473, 82202508, 82350710801, and 82160388), Prevention and Control of Emerging and Major Infectious Diseases‐National Science and Technology Major Project (2025ZD01900700, 2025ZD01904400, and 2026ZD01911700), Yunnan Fundamental Research Project (202401AS070076 and 202403AC100011), and National Key R & D Program of China (2023YFC2306700).

## Ethics Statement

All animal experimental procedures were approved by the Ethics Committee of the Kunming Institute of Zoology (KIZ), Chinese Academy of Sciences (CAS) (approval numbers: IACUC‐PE‐2021‐06‐001 and IACUC‐PE‐2025‐04‐005). All animal experiments complied with the ARRIVE guidelines.

## Conflicts of Interest

The authors declare no conflicts of interest.

## Supporting information




**Supporting File 1**: mco271049‐sup‐0001‐Video S1.mp4

**Supporting File 2**: mco271049‐sup‐0002‐Video S2.mp4

**Supporting File 3**: mco271049‐sup‐0003‐Video S3.avi

**Supporting File 4**: mco271049‐sup‐0004‐Video S4.avi

**Supporting File 5**: mco271049‐sup‐0005‐Video S5.avi

**Supporting File 6**: mco271049‐sup‐0006‐SuppMat.docx

## Data Availability

All data from this study are available from the corresponding author Yong‐Tang Zheng upon reasonable request. Sequencing data supporting the findings of this study have been deposited in the Genome Sequence Archive (GSA) database under accession codes CRA028847 (https://ngdc.cncb.ac.cn/gsa/s/TI6CSmEY), HRA012728 (https://ngdc.cncb.ac.cn/gsa‐human/s/ZqVY217l), and CRA028955 (https://ngdc.cncb.ac.cn/gsa/s/F92p1S5l).
