# Supplementary material for: Polyomavirus Drives Cerebellar Lesions in AIDS Monkeys With Ataxia
Source: MedComm (2020). 2026 Oct 19;7(11):e71049. doi: 10.1002/mco2.71049 (PMC13679839; doi:10.1002/mco2.71049)
Supplement: Supplementary file 1 [file MCO2-7-e71049-s005.docx]

**Polyomavirus Drives Cerebellar Lesions in AIDS Monkeys with Ataxia**

Tian-Zhang Song^1,#^, Li-Rong Zhang^1,2,#^, Yun-Mei Wang^1,#^, Yan Hu^1,3,#^, Qing Li^1^, Ren-Rong Tian^1,4^, Liu-Meng Yang^1^, Si-Dong Xiong^2^, Dong-Dong Wu^1,*^, Yong-Tang Zheng^1,*^

**Affiliations**

1	State Key Laboratory of Genetic Evolution & Animal Models, Key Laboratory of Bioactive Peptides of Yunnan Province, National Resource Center for Non-Human Primates, Kunming Institute of Zoology, Chinese Academy of Sciences, Kunming 650223, China.

2	KIZ-SU Joint Laboratory of Animal Model and Drug Development, College of Pharmaceutical Sciences, Soochow University, Suzhou 215021, China.

3	University of Chinese Academy of Sciences, Beijing 101408, China.

4	Yunnan Provincial Key Laboratory of Entomological Biopharmaceutical R&D, College of Pharmacy, Dali University, Dali 671006, China.

**#These authors contributed equally to this work.**

***Corresponding author**

*Yong-Tang Zheng*, Ph.D

Kunming Institute of Zoology, Chinese Academy of Sciences

No.32, Jiaochang Donglu, Kunming, Yunnan, 650223, China

Tel & Fax: +86 871 65195684. E-mail: zhengyt@mail.kiz.ac.cn

*Dong-Dong Wu*, Ph.D

Kunming Institute of Zoology, Chinese Academy of Sciences

No.17 Longxin Road, Kunming, Yunnan, 650201, China

Tel & Fax: +86 871 65198775. E-mail: wudongdong@mail.kiz.ac.cn

**Supplementary Materials**

***Magnetic resonance imaging (MRI) parameters***

MRI acquisition used the following parameters. For T1-weighted imaging, repetition time (TR) was 14.0 ms, echo time (TE) was 5.5 ms, inversion time (TI) was 940 ms, flip angle was 8°, number of averages was 2, matrix size was 160 × 160 × 150, and voxel size was 0.5 × 0.5 × 0.5 mm^3^. For neurite orientation dispersion and density imaging (NODDI), TR was 7 400 ms, TE was 72.8 ms, flip angle was 90°, b-values were 1 000 g/mm^2^ and 2 000 g/mm^2^, number of averages was 2, matrix size was 96 × 96 × 129, and voxel size was 1.0 × 1.0 ×1.0 mm^3^. Fourteen regions in each cerebellar hemisphere were segmented for analysis based on the D99 atlas. These regions included cerebellar lobules I, II, III, IV, V, VI, VII, IX, and X, superior cerebellar peduncle decussation, superior cerebellar peduncle, inferior cerebellar peduncle, middle cerebellar peduncle, and superficial and deep portions of the pontocerebellar fibers.

***Immunological staining***

Paraffin-embedded sections were incubated with the following primary antibodies: rabbit anti-CD3 (ab16669, Abcam, UK), rabbit anti-CD163 (ab182422, Abcam, UK), rabbit anti-IBA-1 (019-19741, Wako, Japan), rabbit anti-GFAP (80788T, Cell Signaling, USA), rabbit anti-MBP (bsm-61377R, Bioss, China), rabbit anti-NeuN (ab177487, Abcam, UK), rabbit anti-SV40 T antigen (HY-P83511, MCE, USA), and rabbit anti-Ki67 (27309-1-AP, Proteintech, USA). Secondary antibodies included Alexa Fluor 555-conjugated goat anti-rabbit IgG H&L (ab150078, Abcam, UK) and horseradish peroxidase (HRP)-conjugated goat anti-rabbit IgG H&L (BS13278, Bioworld, USA). Tyramine reagents conjugated to iFluor 488 (YT0070L, Uelandy, China), Cy3 (11065, AAT Bioquest, USA), or Cy5 (11061, AAT Bioquest, USA) were used for tyramide signal amplification.

Molecular layer thickness, granular layer cell counts, Luxol fast blue (LFB)-positive white matter area, percentage of abnormal Purkinje cells, and fluorescence intensity were quantified in five randomly selected fields per sample using ImageJ v1.54g (NIH, USA). Microglial morphometry was assessed on IBA-1-immunostained cerebellar sections. Z-stack images (10-µm depth, 0.5-µm intervals) were acquired from the molecular layer, granular layer, and white matter using a STED microscope (Abberior, Germany) with a 40× objective. Ten morphologically intact microglia per layer were randomly selected for analysis. Acquisition and analysis parameters remained consistent across all samples. Consecutive stacks were converted to maximum-intensity projections using STEDYCON software (Abberior, Germany). Images were then denoised and binarized for morphometric analysis in ImageJ.

***Antibodies for flow cytometry***

Anti-human flow cytometry antibodies with cross-reactivity to rhesus macaque antigens included anti-CD3 BV421 (clone SP34-2), anti-CD3 APC-Cy7 (clone SP34-2), anti-CD8 PE-Cy7 (clone PRA-T8), anti-CD8 FITC (clone SK1), anti-HLA-DR BUV661 (clone G46-6), and anti-PD-1 BV605 (clone EH12.1), all purchased from BD Biosciences (USA), and anti-CD4 PerCP/Cy5.5 (clone OKT4) purchased from BioLegend (USA).

***Antibodies for western blot analysis***

Primary antibodies included mouse anti-SV40 T antigen (sc-147, Santa Cruz, USA), rabbit anti-BCL2 (bsm-61074R, Bioss, China), rabbit anti-GSDMD (20770-1-AP, Proteintech, USA), mouse anti-BAX (60267-1-Ig, Proteintech, USA), and rabbit anti-GAPDH (AF7021, Affinity, USA). Secondary antibodies included goat anti-mouse (abs20039, Absin, China) and goat anti-rabbit (abs20040, Absin, China).

***Enzyme-linked immunosorbent assay (ELISA) kits***

Commercial ELISA kits were used to quantify viral antigens, SV40 antibodies, cytokines, and vitamin B1. Viral antigen kits included GY-L0515 for CMV, GY-L1607 for JCV, GY-L1010 for EBV, GY-L1252 for BKV, and GY-L0830 for VZV, all from Guyan (Shanghai, China). The SV40 antibody kit was obtained from Fantai (FT-P1179H, Shanghai, China). Cytokine kits included MM-0139H1 for IL18, MM-0181H1 for IL-1β, MM-0204H1 for IP-10, MM-0049H1 for IL-6, MM-0122H1 for TNF-α, MM-0464H1 for IFN-α, MM-0578H1 for IFN-β, MM-0033H1 for IFN-γ, and MM-0063H1 for IL-12, all from Meimian (Jiangsu, China). The vitamin B1 kit was obtained from mlbio (ml077010, Shanghai, China). Optical density was measured at 450 nm, except for vitamin B1, which was measured at 704 nm, using an ELX800 microplate reader (BioTek, USA).

***RNA sequencing (RNA-seq)***

Total RNA from cerebellar tissues and cell lines was used for RNA-seq. Library construction and sequencing were carried out by Novogene (Beijing, China). In brief, at least 1 μg of RNA per sample was used for RNA sample preparation. Sequencing libraries were generated using a NEBNext^®^ Ultra RNA Library Prep Kit for Illumina^®^ (NEB, USA) according to the recommendations of the manufacturer. Libraries were sequenced on the Illumina sequencing platform (NovaSeq 6000, Illumina, USA). Clean data were obtained by removing reads containing adapters, reads with poly-N content >10%, and reads containing >50% low-quality bases (Q ≤ 5). Reference genomes for rhesus macaque (*Macaca mulatta*; Mmul_10) and human (*Homo sapiens*; GRCh38) were downloaded from Ensembl release 104. DESeq2 was used to assess differential gene expression based on log2 fold change and statistical significance. Differentially expressed genes (DEGs) were defined based on |fold change| > 2 and *P* < 0.05 for cerebellar tissues, and |fold change| > 2 and adjusted *P* < 0.05 for cell lines. Over-representation analysis (ORA) and gene set enrichment analysis (GSEA) were performed using the clusterProfiler R package v4.6.2. Gene sets were obtained from the Gene Ontology Biological Process (GO-BP) and Kyoto Encyclopedia of Genes and Genomes (KEGG) databases, with adjusted *P* < 0.05 used as the significance threshold. Immune-related DEGs were identified using the ImmPort database. Protein-protein interaction (PPI) networks of DEGs and semantic similarity-based connections among GO-BP terms were generated using STRING and REVIGO, respectively.

***Single-nucleus RNA-seq (snRNA-seq)***

**Nuclei isolation and library preparation.** snRNA-seq was performed on cerebellar tissues from seven rhesus macaques, corresponding to the same animals used for bulk RNA-seq. Frozen specimens were minced, homogenized in ice-cold buffer, and filtered through 40-μm strainers. Nuclear release efficiency and integrity were assessed by acridine orange/propidium iodide (AO/PI) staining and a Countstar automated cell counter (Alit Life Science, China). After centrifugation and resuspension in phosphate-buffered saline (PBS) at a final concentration of 1 000–2 000 nuclei/μL, nuclei were loaded into a microfluidic chip from the Chip A Single Cell Kit v2.1 (S050100301, MobiDrop, China) and processed using the MobiNova-100 system (A1A40001, MobiDrop, China) to generate barcoded droplets. Following encapsulation, droplets were subjected to photolytic cleavage using the MobiNovaSP-100 system (A2A40001, MobiDrop, China) to release oligonucleotides into the reaction mixture, enabling mRNA capture by barcoded poly(dT) primers. Reverse transcription was performed within droplets to generate barcoded cDNA. The resulting cDNA was amplified and processed into sequencing libraries using the High Throughput Single-Cell 3′ Transcriptome Kit v2.1 (S050200301, MobiDrop, China) and 3′ Dual Index Kit (S050300301, MobiDrop, China) [1,2]. Final libraries were sequenced on the Illumina NovaSeq 6000 platform, with read 1 and 2 lengths of 40 and 150 bp, respectively.

**Data processing and cell-type annotation.** Raw FASTQ files were aligned to the *Macaca mulatta* genome (Mmul_10, Ensembl v111) using MobiVision v3.0 (MobiDrop, China). Unique molecular identifier (UMI) count matrices were processed in Seurat (v4.4.0) [3,4]. Quality-control filtering was applied at both the gene and nucleus levels. Genes detected in fewer than 20 nuclei were excluded. Nuclei were retained if they contained 200–8 000 detected genes, 1 000–60 000 UMIs, mitochondrial gene content (percent.mt) of ≤1%, and a UMI-to-gene ratio of 1.5–7.5. Filtered data were normalized, scaled, and dimensionally reduced using NormalizeData, ScaleData, and RunPCA functions, respectively. Potential doublets were removed using DoubletFinder with an expected doublet rate of 6% [5]. Seurat count objects from the seven samples were merged and normalized using NormalizeData. S-phase and G2/M scores were calculated with CellCycleScoring using updated cell-cycle markers [6]. Merged data were scaled using ScaleData while regressing out covariates, including sex, age, UMI counts and percent.mt. Batch effects among individual animals were corrected using the Harmony algorithm prior to clustering with FindNeighbors and FindClusters and visualization with RunUMAP. Cluster markers were identified using FindAllMarkers and were defined as genes expressed in ≥25% of cluster cells (min.pct ≥ 0.25), with Bonferroni-adjusted *P* < 0.05 and average fold change (FC) ≥ 1.5. Cell types were annotated using known marker genes and cross-species integration with a reference mouse cerebellar snRNA-seq dataset [7]. Integration was performed using the SelectIntegrationFeatures, FindIntegrationAnchors, and IntegrateData functions in Seurat. One-to-one orthologous genes between human or mouse and rhesus macaque were obtained using BioMart (Ensembl v111). Functional enrichment analysis of marker genes was performed using human gene annotations from org.Hs.eg.db through clusterProfiler v4.6.0 [8].

**Cell-cell communication analysis.** Intercellular communication networks were analyzed in the SIVa, SIVn, and CTL groups using CellChat (v2.1.2) and its human ligand-receptor interaction database, which contains approximately 3 300 interactions across paracrine and autocrine signaling, extracellular matrix (ECM)-receptor, cell-cell contact, and non-protein signaling [9]. Global interaction strength was compared among groups using compareInteractions. Cell-type-specific signaling alterations were assessed by differential communication probability analysis using netAnalysis_diff_signalingRole_scatter, with evaluation of both outgoing (sender) and incoming (receiver) pathways.

**DEG identification.** Pairwise differential expression analysis across all cell types was performed using Seurat FindMarkers for the SIVa versus SIVn, SIVa versus CTL, and SIVn versus CTL comparisons. Genes were considered significantly upregulated in the first group when they were expressed in ≥25% of cells in that group (min.pct ≥ 0.25), with Bonferroni-adjusted *P* < 0.05 and FC ≥ 1.5. Genes were considered significantly downregulated when they were expressed in ≥25% of cells in the second group (min.pct ≥ 0.25), with Bonferroni-adjusted *P* < 0.05 and FC ≤ 1/1.5. Functional enrichment analysis of DEGs was conducted using clusterProfiler.

**References**

1.	Macosko EZ, Basu A, Satija R, et al. Highly parallel genome-wide expression profiling of individual cells using nanoliter droplets. *Cell*. 2015;161(5):1202-1214.

2.	Klein AM, Mazutis L, Akartuna I, et al. Droplet barcoding for single-cell transcriptomics applied to embryonic stem cells. *Cell*. 2015;161(5):1187-1201.

3.	Hao Y, Hao S, Andersen-Nissen E, et al. Integrated analysis of multimodal single-cell data. *Cell*. 2021;184(13):3573-3587.e29.

4.	Wang YM, Wang WC, Pan Y, et al. Regional and aging-specific cellular architecture of non-human primate brains. *Genome Med*. 2025;17(1):41.

5.	McGinnis CS, Murrow LM, Gartner ZJ. DoubletFinder: doublet detection in single-cell RNA sequencing data using artificial nearest neighbors. *Cell Syst*. 2019;8(4):329-337.e4.

6.	Tirosh I, Izar B, Prakadan SM, et al. Dissecting the multicellular ecosystem of metastatic melanoma by single-cell RNA-seq. *Science*. 2016;352(6282):189-196.

7.	Kozareva V, Martin C, Osorno T, et al. A transcriptomic atlas of mouse cerebellar cortex comprehensively defines cell types. *Nature*. 2021;598(7879):214-219.

8.	Wu T, Hu E, Xu S, et al. clusterProfiler 4.0: a universal enrichment tool for interpreting omics data. *Innovation*. 2021;2(3):100141.

9.	Jin S, Guerrero-Juarez CF, Zhang L, et al. Inference and analysis of cell-cell communication using CellChat. *Nat Commun*. 2021;12(1):1088.

**Legend**

Video S1. Postural imbalance in an ataxic macaque.

Video S2. Nystagmus in an ataxic macaque.

Video S3. Whole-brain T1-weighted imaging (CTL).

Video S4. Whole-brain T1-weighted imaging (SIVn).

Video S5. Whole-brain T1-weighted imaging (SIVa).

Table S1. Characteristics of enrolled macaques.

| **Group** | **ID** | **Sex** | **Ataxia** | **Infection** | **Infection duration** | **Viral load (copies/mL)** | | **CD4/CD8** | |
| --- | --- | --- | --- | --- | --- | --- | --- | --- | --- |
|  |  |  |  |  |  | **Peak point** | **Euthanasia** | **Pre-infection** | **Euthanasia** |
| CTL | CTL1 | Male | no | NA | NA | NA | NA | NA | NA |
|  | CTL2 | Male | no | NA | NA | NA | NA | NA | NA |
|  | CTL3 | Female | no | NA | NA | NA | NA | NA | NA |
| SIVn | SIV1 | Male | no | SIVmac239 | 3.5 years | 39187.86 | 72833.61 | 1.39 | 0.28 |
|  | SIV2 | Male | no | SIVmac239 | 3.5 years | 16315.02 | 3473.65 | 1.36 | 0.62 |
| SIVa | SIV3 | Female | yes | SIVmac239 | 3.5 years | 30859.98 | 20351.29 | 2.44 | 0.52 |
|  | SIV4 | Female | yes | SIVmac239 | 3.5 years | 173556.39 | 80438.68 | 1.51 | 0.14 |

Table S2. List of PCR primers and probes.

| **Gene** | **Sequence** |
| --- | --- |
| SIV (Forward primer) | 5'-TCGGTCTTAGCTCCATTAGTGCC-3' |
| SIV (Reverse primer) | 5'-GCTTCCTCAGTGTGTTTCACTTTC-3' |
| SIV (Probe) | 5'-CTTCTGCGTGAA TGCACCAGATGACGC-3' |
| RPL13A (Forward primer) | 5'-GGCTAAACAGGTACTGCTGGG-3' |
| RPL13A (Reverse primer) | 5'-AGGAAAGCCAGGTACTTCAACTT-3' |
| ISG15 (Forward primer) | 5'-GCAGTTGAGAGGCAGTGAAC-3' |
| ISG15 (Reverse primer) | 5'-TTCAGGTCCCAGCTCATGG-3' |
| MX2 (Forward primer) | 5'-AATCGTAACCAGGTGTCCGC-3' |
| MX2 (Reverse primer) | 5'-TGACGTTCTGGGCTTTGTGT-3' |
| DHX58 (Forward primer) | 5'-ATGGAGCTGCGGCCCTAC-3' |
| DHX58 (Reverse primer) | 5'-ACCAGGTGCACCCTGTTGA-3' |
| IFIT3 (Forward primer) | 5'-AGAGCAGTCATGAGTGAGGTC-3' |
| IFIT3 (Reverse primer) | 5'-CACACTCTGTCTTCCAGATCCC-3' |
| IRF9 (Forward primer) | 5'-CTTATCACAGTGAAGATGGAGCA-3' |
| IRF9 (Reverse primer) | 5'-AAGAGGACAGGTCAATCGTG-3' |
| CXCL10 (Forward primer) | 5'-TTGCTGCCTTGTCTTTCTGAC-3' |
| CXCL10 (Reverse primer) | 5'-GACACCTCTTCTCACCCTTCTTT-3' |
| CCL2 (Forward primer) | 5'-TTCTGTGCCTGCTGCTCATAG-3' |
| CCL2 (Reverse primer) | 5'-GGGGGCATTGATTGCATCTGG-3' |
| CXCL8 (Forward primer) | 5'-GAAACCACCGGAAGGAACCA-3' |
| CXCL8 (Reverse primer) | 5'-TTGGCAAAACTGCACCTTCAC-3' |
| OAS2 (Forward primer) | 5'-TCCAGATTGTCCAGGGAGGA-3' |
| OAS2 (Reverse primer) | 5'-ATGGAACACGACGAGGTTGG-3' |
| STAT1 (Forward primer) | 5'-ACTCTGTGCATGCAACCTCG-3' |
| STAT1 (Reverse primer) | 5'-ATACCACTGAGACATCCTGCC-3' |

**Figure S1. Bulk RNA sequencing and cytokine profiling.** **(A)** Over-representation analysis (ORA) of the top 10 upregulated Gene Ontology Biological Process (GO-BP) terms in the SIVa versus CTL comparison (*n_CTL/SIVa_* = 3/2; terms ranked by adjusted *P* value). Red labels indicate immune- and defense-related pathways. **(B)** Left: Venn diagram showing overlap of upregulated immune-related differentially expressed genes (DEGs) among three pairwise comparisons (SIVn versus CTL, SIVa versus CTL, and SIVa versus SIVn). Right: merged protein-protein interaction (PPI) network of upregulated immune-related DEGs from the three comparisons, showing the top 30 genes ranked by degree and constructed using the STRING database. Nodes are color-coded according to the categories shown in the Venn diagram. **(C)** Cytokine levels in plasma, cerebrospinal fluid (CSF), and cerebellar tissue homogenates (*n_CTL/SIVn/SIVa_* = 2/2/2). **(D)** Gene set enrichment analysis (GSEA) of downregulated pathways. Top 10 significantly enriched GO-BP terms in the SIVa versus CTL and SIVn versus CTL comparisons are shown, ranked by normalized enrichment score (NES). Blue labels indicate neuro-related pathways.

**Figure S2. Histological staining of cerebellar tissues and quantitative analysis of MBP expression.** **(A, B)** Cerebellar sections from CTL and SIVn groups were analyzed by (A) periodic acid-Schiff (PAS) staining and (B) Gram staining. ML, molecular layer; GL, granular layer; WM, white matter. **(C)** Quantitative analysis of relative myelin basic protein (MBP) expression levels in SV40‑positive versus SV40‑negative regions in the ataxic cerebellum. ***P* < 0.01.

**Figure S3. SV40 serostatus and peripheral T-cell phenotypes.** **(A)** SV40 antibody levels in pre-infection and pre-necropsy plasma samples measured by ELISA (*n_CTL/_n_SIVn/SIVa_* = 3/2/2). **(B)** Flow cytometric analysis of T-cell activation (HLA-DR^+^) and exhaustion (PD-1^+^) in peripheral CD4^+^ and CD8^+^ T cells (*n_CTL/_n_SIVn/SIVa_* = 3/2/2).

**Figure S4. Cellular characterization of the cerebellum by single-nucleus RNA sequencing. (A, B)** UMAP visualization showing dataset integration across all seven samples (A) and the three experimental groups (B), with minimal batch effects. **(C)** Cross-species alignment with a reference mouse cerebellar single-cell atlas showing conserved cell-type organization after mapping through reciprocal one-to-one orthologs. **(D, E)** Bar plots summarizing the distribution of nuclei across cell types (D) and groups (E). **(F, G)** Distribution of S/G2-M scores (F) and sample composition (G) across cell types, showing that the proliferative state was concentrated primarily in the cycling granule cell subtype and that sample variation remained consistent within groups.

**Figure S5. Comparative analysis of intercellular communication. (A)** Bar plots showing inferred interaction number (top) and interaction strength (bottom) across the three groups. **(B)** Differential signaling roles of individual cell populations. Positive values indicate increased outgoing (sender) or incoming (receiver) signaling capacity in the latter group of each comparison (right of “vs.”), whereas negative values indicate reduced signaling capacity.

**Figure S6. Cytokine secretion by SV40-infected SH-SY5Y cells.** Levels of pro-inflammatory cytokines in supernatants from SH-SY5Y cells infected with SV40 (MOI = 0.01), measured at 0, 12, and 24 hours post-infection (hpi). Data are presented as mean ± SD. ns, not significant; **P* < 0.05.

**Figure S7. Immunofluorescence analysis of cerebellar sections.** **(A–C)** Representative images showing (A) CD3 staining (white arrows: CD3^+^ cells), (B) co-staining of CD163 and IBA-1, and (C) GFAP staining. In (B), IBA-1^+^CD163^-^ cells with ramified morphology were identified as resting microglia; IBA-1^+^CD163^+^ cells were identified as activated microglia; and cells with round or ameboid morphology and an IBA-1^-^CD163^+^ phenotype were identified as infiltrating macrophages (white arrows). (D) Quantification of GFAP^+^ mean fluorescence intensity (MFI) across cerebellar layers. Each data point represents the mean value from five randomly selected fields per animal.

**Figure S8. RNA-seq analysis of SV40-infected HMC3 cells. (A)** GO-BP enrichment analysis of upregulated DEGs. Top 10 significantly enriched terms are shown, ranked by adjusted P value. Terms related to immune and defense responses are highlighted in red. **(B)** PPI network of upregulated DEGs. Node color intensity indicates degree, with darker color corresponding to higher connectivity.
